# Construction of a high-resolution genetic linkage map and comparative genome analysis for the reef-building coral *Acropora millepora*

**DOI:** 10.1186/gb-2009-10-11-r126

**Published:** 2009-11-10

**Authors:** Shi Wang, Lingling Zhang, Eli Meyer, Mikhail V Matz

**Affiliations:** 1 Section of Integrative Biology, School of Biological Sciences, University of Texas at Austin, 1 University Station C0930, Austin, TX 78712, USA

## Abstract

**Background:**

Worldwide, coral reefs are in decline due to a range of anthropogenic disturbances, and are now also under threat from global climate change. Virtually nothing is currently known about the genetic factors that might determine whether corals adapt to the changing climate or continue to decline. Quantitative genetics studies aiming to identify the adaptively important genomic loci will require a high-resolution genetic linkage map. The phylogenetic position of corals also suggests important applications for a coral genetic map in studies of ancestral metazoan genome architecture.

**Results:**

We constructed a high-resolution genetic linkage map for the reef-building coral *Acropora millepora*, the first genetic map reported for any coral, or any non-Bilaterian animal. More than 500 single nucleotide polymorphism (SNP) markers were developed, most of which are transferable in populations from Orpheus Island and Great Keppel Island. The map contains 429 markers (393 gene-based SNPs and 36 microsatellites) distributed in 14 linkage groups, and spans 1,493 cM with an average marker interval of 3.4 cM. Sex differences in recombination were observed in a few linkage groups, which may be caused by haploid selection. Comparison of the coral map with other metazoan genomes (human, nematode, fly, anemone and placozoan) revealed synteny regions.

**Conclusions:**

Our study develops a framework that will be essential for future studies of adaptation in coral and it also provides an important resource for future genome sequence assembly and for comparative genomics studies on the evolution of metazoan genome structure.

## Abstract 2

A high-resolution genetic linkage map for the coral Acropora millepora is constructed and compared with other metazoan genomes, revealing syntenic blocks.

## Background

Although substantial effort is being devoted to understand physiological mechanisms of coral stress tolerance and acclimation [1-3], virtually nothing is currently known about the mechanisms that might enable their adaptation to the changing climate over generations. We have recently demonstrated that the coral *Acropora millepora *shows considerable genetically determined variation in thermal tolerance and responsiveness of the larvae to the settlement cue, which may be the raw evolutionary material for future local thermal adaptation or modification of the larval dispersal strategy in response to ongoing climate change [4]. A high-resolution genetic linkage map would enable identification of the quantitative trait loci (QTLs) associated with these and other adaptation-relevant physiological traits [5,6]. To date, however, no genetic map has been constructed for any coral species, mainly due to lack of genetic resources for most corals.

The coral *A. millepora*, like the majority of hermatypic (algal symbiont-hosting) corals of the order Scleractinia, is a diploid hermaphrodite with 2n = 28 chromosomes [7]. *A. millepora *is common across the Indo-Pacific. As a representative of the most speciose and ecologically important coral genus *Acropora*, *A. millepora *is becoming the leading coral model in terms of molecular groundwork. Currently, 50 microsatellite markers are available for this species [8,9]. Although these markers are obviously not enough for linkage mapping, they are already the largest marker collection available for any reef-building coral. Single nucleotide polymorphisms (SNPs) are the most abundant type of genetic variation in eukaryotic genomes, and are the preferred genetic markers for a variety of applications such as high-resolution linkage mapping, QTL mapping of complex traits, and for combining these results with population genomics, which is arguably the most powerful way of detecting and understanding the process of natural adaptation [10]. Previously, our group has released a large body of sequence data for *A. millepora *obtained by 454 sequencing of the larval transcriptome [11]. More than 33,000 putative SNPs have been identified in these data. Since the detected SNPs reside in or immediately next to the protein-coding sequences ('gene-based SNPs'), they are particularly useful for QTL mapping and population genomics studies because they have the potential for quickly identifying causal genes underlying complex traits [12,13].

A genetic linkage map, especially gene-based, is also an excellent platform for comparative genome studies. Recent comparative genome analyses based on genetic maps have already provided new insights into genome organization, evolution, and function across different organisms [14-20]. For example, comparison of the *Caenorhabditis briggsae *genetic map and the *Caenorhabditis elegans *genome reveals extensive conservation of chromosome organization and synteny despite a very long divergence time (80 to 110 million years), suggesting that natural selection operates at the level of chromosomal organization [14]. In another study, a genetic linkage map of the blind Mexican cavefish *Astyanax mexicanus *has been successfully applied to predict candidate quantitative trait genes relating to rib number and eye size by anchoring cavefish QTLs to the zebrafish genome [16]. The phylum Cnidaria is the sister group of the Bilateria. Anthozoan cnidarians such as corals are phylogenetically basal in the phylum Cnidaria, and have proven to be particularly informative for understanding the evolution of metazoan genetic and developmental complexity [21,22]. Identification of conserved synteny blocks across coral and other metazoan genomes would help to unravel ancestral metazoan genome architecture.

Here, we report the first high-resolution genetic linkage map for a reef-building coral, *Acropora millepora*, which was constructed based on a family of larvae from a cross between two naturally heterozygous coral individuals from Magnetic Island, Australia (an outbred full-sib cross design). An investigation of SNP transferability was carried out in two more populations. Sex differences in recombination were observed in the coral linkage map. Comparison of the coral map with other metazoan genomes (human, nematode, fly, anemone and placozoan) was conducted to identify syntenic regions. This coral genetic map should lay a solid foundation for a variety of future genetic and genomic studies such as QTL mapping of adaptive traits, population genomics, comparative genomics, and assembly of the coral genome.

## Results

### SNP marker development

For SNP marker development, we designed PCR primers for 1,033 candidate SNPs, which were previously identified in the *A. millepora *larval transcriptome by 454-FLX sequencing [11]. After PCR amplification, 603 produced single strong bands with expected sizes, of which 427 SNPs were heterozygous in at least one parent of the mapping family, 91 were homozygous in both parents but for two different alleles, and 85 showed no genetic variations in two parents. Although we restricted the expected amplicon length to about 100 bp in primer design, 208 primer pairs still produced single strong bands but of larger than expected sizes, indicating potential introns in the vicinity of the SNPs. Longer amplicons greatly diminish the precision of high-resolution melting (HRM) SNP analysis, so most of these intron-containing amplicons were discarded. Only four SNP markers developed based on intron sequences were included in this study. The remaining 222 attempted SNP assays resulted in poor amplification (very little or no product) or bad melting curves, suggesting non-specific amplification.

In order to evaluate the transferability of our markers in other populations of *A. millepora*, we randomly selected 48 SNP markers to test their applicability on 7 colonies from 2 Australian Great Barrier Reef locations, Orpheus Island (n = 4) and Great Keppel Island (n = 3), which are 80 km and 570 km away from Magnetic Island, respectively. All the 48 SNP markers could be successfully amplified in the assayed samples. Notably, 36 (75%) and 31 (65%) of them were still polymorphic in the Orpheus Island and Great Keppel Island populations, respectively, despite the fact that only a few individuals were assayed.

### Linkage mapping

Linkage analysis was carried out using JoinMap 4.0 software [23]. In total, 469 markers (431 SNPs and 38 microsatellites) were heterozygous in at least one parent of the mapping family, and were therefore included in the linkage analysis. Segregation analysis showed that 380 markers conform to the expected Mendelian ratios at *P *≥ 0.05 level. More than half of the distorted markers depart only slightly from expected Mendelian ratios (0.01 <*P *< 0.05).

**Figure 1:**
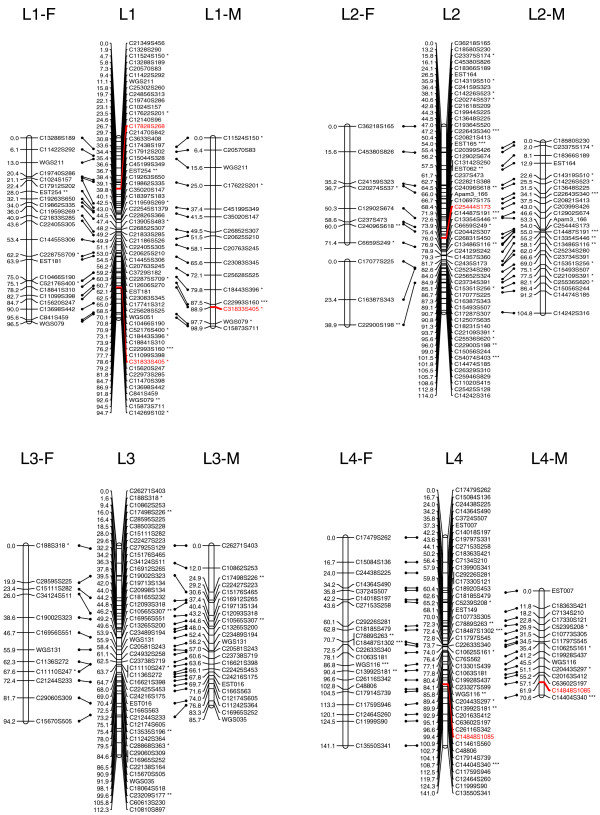
A genetic linkage map (L1 to L4) of the reef-building coral .  Female (F) and male (M) maps are shown on the left and right, respectively, and the consensus map is shown in the center. Homologous loci are connected with solid lines. Putative stress-related markers are shown in red. Distorted loci are indicated by asterisks (*0.01 <*P *< 0.05, ** *P *< 0.01; *** *P *< 0.001).

**Figure 2:**
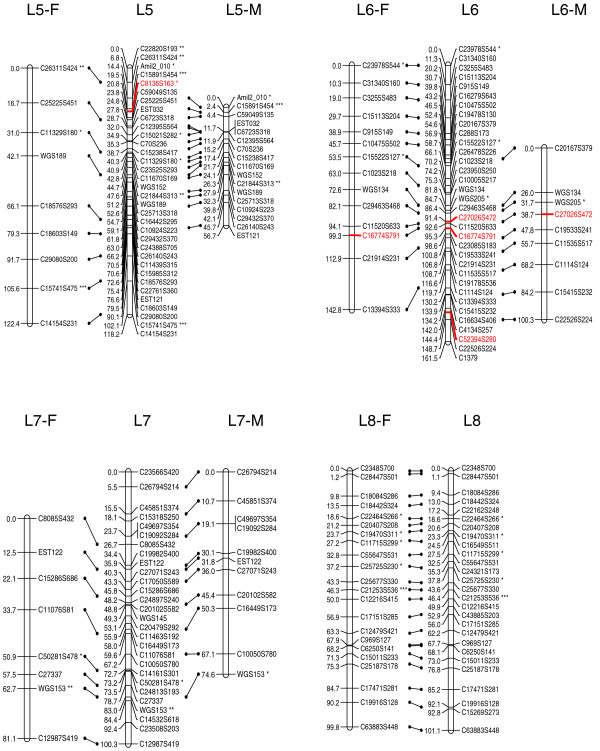
A genetic linkage map (L5 to L8) of the reef-building coral *A. millepora.  *Female (F) and male (M) maps are shown on the left and right, respectively, and the consensus map is shown in the center. Homologous loci are connected with solid lines. Putative stress-related markers are shown in red. Distorted loci are indicated by asterisks (*0.01 <*P *< 0.05, ** *P *< 0.01; *** *P *< 0.001).

**Figure 3:**
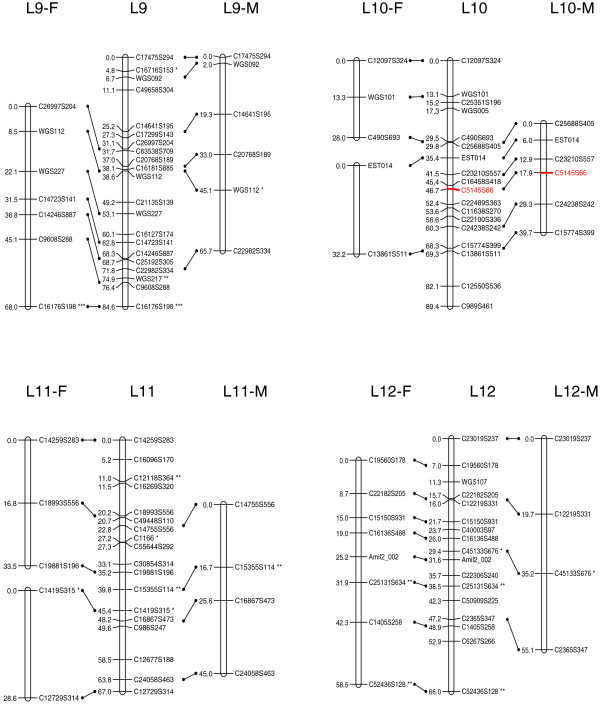
A genetic linkage map (L9 to L12) of the reef-building coral *A. millepora.  *Female (F) and male (M) maps are shown on the left and right, respectively, and the consensus map is shown in the center. Homologous loci are connected with solid lines. Putative stress-related markers are shown in red. Distorted loci are indicated by asterisks (*0.01 <*P *< 0.05, ** *P *< 0.01; *** *P *< 0.001).

**Figure 4:**
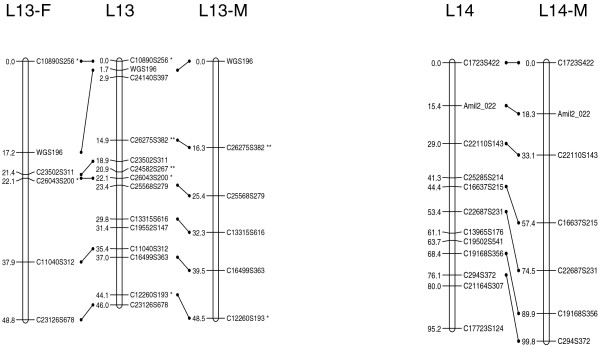
A genetic linkage map (L13 and L14) of the reef-building coral *A. millepora.  *Female (F) and male (M) maps are shown on the left and right, respectively, and the consensus map is shown in the center. Homologous loci are connected with solid lines. Distorted loci are indicated by asterisks (*0.01 <*P *< 0.05, ** *P *< 0.01; *** *P *< 0.001).

At the initial logarithm of the odds (LOD) threshold of 4.5, 293 markers were grouped into 14 linkage groups, which corresponds to the known haploid chromosome number for this species. Then 124 markers were added to the established groups at LOD = 3, and 14 additional markers were added at LOD = 2.5. After data partitioning by the Joinmap 4.0 program, the maternal (1:1 female type) and paternal (1:1 male type) datasets contained 167 and 155 markers, respectively, which were subsequently used for constructing sex-specific maps based on the two-way pseudo-testcross strategy [24]. The female map contains 152 markers and spans 1,185.8 cM, while the male map contains 149 markers and spans 945.4 cM (Figures 1, 2, 3 and 4). The female map is 240.4 cM (30%) longer than the male map, even discounting L8 and L14 where recombination information is missing for one parent. Large differences between recombination rates in the male and female parents were observed for linkage groups L4, L5, L6, L10 and L11 (Table 1). Notably, we found that the polymorphism level revealed by markers in L8 was significantly lower than the average in the male parent (chi-square test, *P *< 0.0001). More interestingly, we found that more than half of the annotated genes in this linkage group were putatively involved in sexual reproduction (Table 2).

**Table 1:** Summary of the coral genetic linkage map

Linkage group	Number of markers	Length (cM)	Average marker interval (cM)	Length in female map (cM)	Length in male map (cM)	Ratio of female/male recombination rate
1	59	94.7	1.6	96.5	98.9	1.0
2	57	114.0	2.0	110.3	104.8	1.1
3	46	112.3	2.5	94.2	85.7	1.1
4	44	141.0	3.3	141.1	70.6	2.0
5	34	118.2	3.6	122.4	56.7	2.2
6	34	161.5	4.9	142.8	100.3	1.4
7	28	100.3	3.7	81.1	74.6	1.1
8	27	101.1	3.9	99.8	NA	NA
9	21	84.6	4.2	68.0	65.7	1.0
10	18	89.4	5.3	60.2	39.7	1.5
11	18	67.0	3.9	62.1	45.0	1.4
12	17	66.0	4.1	58.5	55.1	1.1
13	14	46.0	3.5	48.8	48.5	1.0
14	12	95.2	8.7	NA*	99.8	NA
All	429	1,391.0	3.4	1,185.8	945.4	1.3

*Not available (NA) due to the lack of recombination information for one of the parents.

**Table 2:** A list of genes from linkage group 8 that are putatively involved in sexual reproduction

Marker	Position (cM)	Gene name	Biological process	Reference
C2348S700	0	Tubulin-specific chaperone A (TBCA)	Spermatogenesis	[94]
C20407S208	20.6	Death-associated protein kinase 3 (Dapk3)	Spermatogenesis	[95]
C19470S311	23.3	RNA-binding protein MEX3C (Mex3c)	Regulation of germ cell mitosis	[96]
C16549S511	24.5	Myosin-13 (MYH13)	Oogenesis	[97]
C21253S536	46.4	Zinc finger RNA-binding protein (ZFR)	Meiosis I	[98]
C12216S415	49.9	Translocon-associated protein subunit beta (Ssr2)	Spermatogenesis	[99]
C43885S203	52.9	Chromodomain-helicase-DNA-binding protein 1 (CHD1)	Gametogenesis	[100]
C12479S421	62.2	Putative tyrosinase-like protein tyr-1 (tyr-1)	Spermatogenesis	[101]
C6250S141	68.1	Zinc finger CCHC domain-containing protein 9 (ZCCHC9)	Spermatogenesis	[102]
C15011S233	73.0	Serine protease 23 (PRSS23)	Ovary remodeling	[103]
C25187S178	76.8	SNARE-associated protein Snapin (Snapin)	Spermatogenesis	[104]
C63883S448	101.1	WD repeat-containing protein 47 (Wdr47)	Spermatogenesis	[101]

The consensus map contains 429 markers (393 SNPs and 36 microsatellites) in 14 linkage groups (Figures 1, 2, 3 and 4), and spans 1,391 cM with an average marker interval of 3.4 cM. The length of each linkage group ranges from 46 cM to 161.5 cM. Marker density varies dramatically across linkage groups (Table 1). For example, both L1 and L14 are approximately 95 cM in length, but L1 contains 59 markers whereas L14 contains only 12 markers. Nine putative stress-related genes were identified in the consensus map (Figures 1, 2 and 3; EM and MVM, unpublished) [25,26]. These genes are involved in cytoskeleton formation, heat shock, oxidative stress, protein degradation, and vesicular transport.

Genome lengths estimated by two different methods [27,28] are similar at 1,484.8 cM (*G*_*e*1_) and 1,501.9 cM (*G*_*e*2_), respectively. The average of two estimates was taken as the expected genome length - 1493.4 cM. Given an estimated genome size of 200 Mbp for *A. millepora *[1], the average recombination rate across all linkage groups is approximately 7.5 cM/Mbp. The genome coverage of the current map was estimated as 93.1%.

### Comparative genome analysis

Comparison of the markers mapped in this study with the previously annotated coral larval transcriptome [11] allowed the assignment of nearly all markers (97%) to longer cDNA sequences, which included all markers derived from 454 transcriptome sequences. Of the 416 sequences so identified, 286 (69%) corresponded to known genes based on the previously described transcriptome annotation [11]; 280 genes mapped by this process were each associated with a single marker, with 6 genes containing two markers each. The accession numbers, gene annotation, and synteny information for all mapped markers are shown in Additional data file 1.

**Figure 5:**
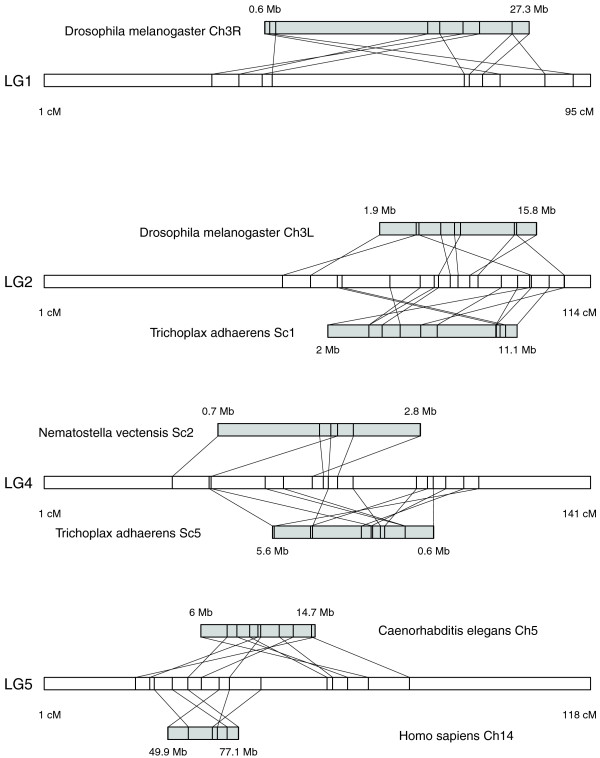
Conserved synteny blocks.  Each synteny block represents a set of mapped coral markers and their best matches in another metazoan genome. Synteny blocks were defined as groups of at least three markers, each of which was ≤10 cM from its nearest neighbor within a linkage group in the coral map, and for which the best matches in another genome were also each ≤10 Mb from their nearest neighbors in the same chromosome or scaffold. All blocks shown here contain more markers than expected from chance (*P *< 0.05) based on permutation analysis (n = 1,000). For each block, the coral linkage group is shown as a white horizontal bar, with syntenic marker positions (in cM) indicated on the bar. For each linkage group containing a synteny block, each comparison with the other genome is shown as a horizontal grey bar, with marker positions (in Mb) indicated on the bar. Relationships between coral markers and other genomes, based on sequence similarity (tblastx, bit-score ≥50), are indicated by diagonal lines connecting each coral marker with its best match.

Comparison of the mapped sequences with assembled genomes from other metazoans identified putative homologs for between 48% (nematode) and 80% (sea anemone) of the mapped coral genes, and a similar comparison with the yeast genome identified putative homologs for 29% of mapped coral genes. These pairs of putative homologs allowed for comparison of the coral genetic map with assembled genome sequences of other metazoans, identifying conserved synteny blocks in 11 of the 14 coral linkage groups, each of which contained from 3 to 12 markers. The largest synteny block conserved between coral and another metazoan was found in linkage group 4, with 12 markers spanning 69 cM in the coral linkage group and their best matches spanning 5 Mb in scaffold 5 of the *Trichoplax adhaerens *genome (Figure 5). An overlapping set of markers within this same linkage group also showed conserved synteny with the anemone *Nematostella vectensis *(Figure 5). Synteny blocks were identified in each of the metazoan comparisons; each comparison identified 4 to 13 blocks, with each block containing 3 to 12 markers (Table 3). Most of the conserved synteny blocks identified here involved intra-chromosomal rearrangements, in which linkage was conserved but gene order was not (for example, the synteny block conserved between coral and placozoan in Figure 5). Notably, a parallel comparison between the coral map and the yeast genome found no evidence of conserved synteny, even though the small genome size of yeast (approximately 12 Mb) would be expected to relax one of the operational criteria for determining synteny (the requirement that matches occur within ≤10 Mb in the yeast chromosome).

**Table 3:** Synteny blocks between *A. millepora *and other eukaryotic genomes and their significance

	All synteny blocks			Significant blocks	
			
Comparison	Blocks (n)	Markers per block	Overall significance	Blocks (n)	Markers per block
*Saccharomyces cerevisiae*	0	0	1	0	0
*Homo sapiens*	4	3-6	0.002	1	6
*Nematostella vectensis*	6	3-6	< 0.001	1	6
*Caenorhabditis elegans*	12	3-10	0.392	1	10
*Drosophila melanogaster*	13	3-10	0.679	2	9-10
*Trichoplax adhaerens*	13	3-12	0.002	2	12

Overview of synteny blocks identified by comparisons between the genetic map of *A. millepora *and other eukaryotic genome sequences, with permutation tests to evaluate significance of synteny blocks. Probabilities were based on permutation tests, as described in Materials and methods. The *P*-value reported for overall significance reflects the likelihood that the observed number of conserved synteny blocks would be expected by random chance. Significance of synteny block sizes was based on the likelihood that a block containing at least that many markers would be expected by chance.

We tested for the significance of synteny blocks using randomly shuffled permutations of the original data, which revealed that a non-trivial number of synteny blocks could be expected to emerge in these comparisons by random chance (Table 3). Although numerous synteny blocks were detected in comparisons between coral and *Drosophila melanogaster *or *C. elegans*, the number of blocks detected was not significantly higher than expected by chance for either comparison (*P *= 0.68 and *P *= 0.39, respectively). In contrast, the other three metazoan genomes we investigated each showed significantly more synteny than expected by chance (anemone, *P *< 0.001; placozoan, *P *= 0.002; human, *P *= 0.002). Obviously the comparison with yeast (*Saccharomyces cerevisiae*), which found no conserved synteny, was unaffected by these statistical tests. Each of the metazoan genome comparisons identified at least one synteny block that contained more markers (n = 6 to 12) than expected by chance. These significant blocks of conserved synteny are depicted in Figure 5, and the syntenic markers in each block are described in more detail in Additional data file 1.

## Discussion

### SNP marker development in coral

Molecular markers are useful tools for assessing important ecological and evolutionary issues such as connectivity, local adaptation, range shifts, biodiversity depletion, speciation, and invasion. Despite widespread concerns about the future of reef-building corals in the changing climate, genetic resources for corals remain scarce. The traditional ways of developing microsatellites or SNP markers are quite costly and time-consuming. Moreover, due to technical problems and low abundance in the genome, it has been shown that development of a large number of microsatellite markers in acroporid corals is particularly difficult based on the traditional microsatellite-enriched genomic library method [29]. Despite the advantages of SNP markers for a variety of tasks [30], their use in non-model organisms such as corals has been hampered primarily due to the costs of high-throughput SNP discovery and genotyping. With the introduction of the next-generation 454 sequencing technology, high-throughput SNP discovery is now feasible for any non-model organism. Our previous study [11], as well as others recently published [31-33], demonstrates a cost-effective way to produce a large number of gene-associated SNPs from transcriptome data obtained by 454 sequencing. Such gene-derived SNPs are particularly useful for non-model organisms, since they stand a better chance of identifying causal genes underlying complex traits in these organisms in the absence of genome sequence data [12,13]. The criteria that we used for SNP mining (at least 3× occurrence of the minority allele and at least 6× read coverage) are more stringent than those typically used (2× occurrence of the minority allele, and 4× or 5× read coverage) [11,31,32]. In our experience, the use of these stringent criteria enhances the success rate of marker development from 454 sequencing data.

### SNP genotyping via high resolution melting analysis

Among the methods available for high-throughput SNP genotyping, the simple, fast and cost-effective HRM method is especially suitable for non-model organisms. The original HRM method requires one fluorescently labeled probe for each assay [34,35]. Later, this method was simplified by using an unlabeled probe in the fluorescent dye solution, but the 3' end of the probe still required costly chemical modification to prevent extension of the probe [36,37]. In this study, we further decrease HRM genotyping cost simply by adding two mismatched bases to the 3' end of an unlabeled probe instead of chemical modification.

### SNP marker transferability between populations

Transferability of the assays to different populations is arguably the most important problem that may arise when trying to apply SNP markers to broad-scale population studies. The markers developed for one population may turn out to be appreciably polymorphic only in populations well connected to the original one, while being essentially homozygous in other, more isolated populations. The degree of connectivity between *A. millepora *populations between three reefs in the Great Barrier Reef (representing northern, middle, and southern regions) has been previously evaluated using allozyme markers [38]. Similar to nearly all coral species in that analysis, *A. millepora *demonstrated genetic subdivision among sampled sites (high F_st _values), although not without some connectivity (an estimated 5 to 30 exchanged migrants per generation). Oliver and Palumbi [39], on the other hand, detected strong barriers to connectivity over longer spatial scales (across Pacific archipelagoes) in two closely related species, *A. cytherea *and *Acropora hyacinthus*, using several intron- and mitochondrial DNA-derived markers that were developed for phylogeography applications. The study of the natural genotypic diversity and connectivity between *A. millepora *populations is of great interest for understanding the evolutionary responses of reef-building corals to ongoing climate change, and is among our high-priority research areas for the future. This emphasizes the importance of determining whether our SNP markers are polymorphic in other populations, or mostly represent 'private alleles' specific to the Magnetic Island (and perhaps even more specifically, Nelly Bay) population. Fortunately, in our interpopulation transferability test, most (65 to 75%) of the SNP markers we tested were polymorphic in just seven *A. millepora *colonies from Orpheus Island and Great Keppel Island, which are 80 km and 570 km away from Magnetic Island, respectively. Although this result suggests that the detected SNPs represent relatively common alleles in these populations, the distance between these populations is just a fraction of what was assayed in the Ayre and Hughes study [38], and so it remains to be seen how far this allele sharing extends. Still, this result is quite promising and suggests the potential for application of these SNP markers to inter-population studies of local adaptation in *A. millepora*.

### Mapping population

For animals and plants with short generation times, very efficient mapping populations (second generation (F_2_), backcross, recombinant inbred lines, double haploid, and so on) can be generated from the crosses among homozygous paternal strains or recombinant inbred lines, which usually requires multiple generations of sib-mating or self-fertilization. Despite several advantages of those methods, it would be very difficult, if not impossible, to produce such mapping populations in corals because most corals have long generation times (approximately 5 to 10 years in some corals, and 3 to 5 years in most acroporids), and the adult colonies are rather difficult to maintain. Last but not least, to our knowledge, synchronized coral mass spawning, an essential requirement for making genetic crosses, has never been recreated in laboratory-raised corals. In short, corals make poor laboratory models; however, this does not diminish the value of ecological and evolutionary questions pertaining to these organisms. Fortunately, previous studies have shown that *A. millepora*, like many other corals, is a highly heterozygous species [8,9]. Because of this, an outbred full-sib family would be a suitable mapping population for constructing a linkage map [40-45]. Although marker configurations are more complicated in such a family, they can be deduced after analyzing the parental origin and genetic segregation of the markers in the progeny (for a review, see [46]). In particular, coral larvae offer several key advantages over adult colonies for linkage mapping in that they are easy to obtain in great numbers, and, in this species, they do not contain algal symbionts, which would be a potential source of DNA contamination.

### Map density and recombination rate

In the consensus map, marker density is dramatically variable across linkage groups, indicating that the protein-coding genes in *A. millepora*, like in human [47], are distributed very unevenly among chromosomes. This also suggests that including anonymous genetic makers into the current map will likely increase marker density in less populated linkage groups. The current genetic map covers 93% of the *A. millepora *genome and has a resolution of 3.4 cM, which should be sufficient for QTL mapping [48,49]. The average recombination rate across all linkage groups is approximately 7.5 cM/Mb in *A. millepora*, which is much higher than human (1.20 cM/Mb [50]), mouse (0.5 cM/Mb [50]), *D. melanogaster *(2 cM/Mb [51]), and even the plant *Arabidopsis thaliana *(5 cM/Mb; calculated based on data from The Arabidopsis Information Resource website [52]). This suggests that QTLs, if identified, can be narrowed down to rather small genomic regions in this coral species. Nine putative stress-related genes were mapped in the consensus map (markers colored red in Figures 1, 2 and 3), and it would be interesting to see whether any of these are highlighted in future QTL mapping of adaptive physiology traits, such as heat tolerance. Moreover, SNPs in these genes might also prove useful for the study of allele-specific gene expression [53]. Last but not least, the high-resolution genetic linkage map would be invaluable for assembling the *A. millepora *genome, the sequencing of which is imminent (DJ Miller, personal communication).

### Gamete-specific recombination rates

Differential recombination rates between sexes are widespread in animals and plants, with females often having more recombination and longer genetic maps than males [54]. Similar observations have also been reported in hermaphrodites, with greater recombination in female than male gametic tissue [41,55,56]. The underlying mechanism remains the subject of much debate, although several models have been proposed (for a review, see [57]). In this study, the length of the female map is 30% longer than that of the male map, suggesting that sex difference in recombination does exist in *A. millepora*. However, this difference seems attributable to only a few (that is, L4, L5, L6, L10 and L11), but not all, linkage groups. The 'haploid selection' model proposed by recent studies [58,59] seems to be the most plausible explanation for our observation. In the 'haploid selection' theory, sex differences in recombination result from a male-female difference in gametic selection. In coral *Acropora *spp., like in most animals, there is no female haploid phase, because meiosis is completed only after fertilization [60]. Since some genes (for example, genes responsible for meiotic drive systems) are expressed and under selection during the male haploid phase [61,62], this would tend to reduce recombination in males. If such genes were located in only a few chromosomes, this would be expected to reduce the amount of recombination observed in those chromosomes.

Haploid selection might also explain the low polymorphism level of linkage group 8 in the male parent. Because the male parent was genotyped based on the sperm sample, it is possible that genotypes of some loci inferred from sperm mixtures are different from genotypes of adult tissues if these loci are subject to haploid selection. The significant low polymorphism level in L8 of the male parent may reflect strong haploid selection (for example, one of the homologous chromosomes corresponding to L8 might produce functional sperm, while the other might contain deleterious alleles that would produce non-functional sperm). Direct validation of this hypothesis would require tissue samples from the male parent, which are not available. However, the finding that more than half of annotated genes in L8 have putative roles in sexual reproduction supports the idea that this linkage group may be a target for haploid selection.

### Synteny analysis and permutation tests

Synteny is defined as consistent linkage between certain genes across species. In the most general case, the definition does not require conservation of gene order or orientation. Previous comparative genomics studies have revealed synteny between distantly related metazoan taxa [63,64]. Most studies of genome evolution in animals have focused on bilaterian taxa for which extensive genomic resources are available [16,65-67]. More recently, the draft assemblies of the sea anemone and placozoan genomes have revealed substantial synteny between more distantly related metazoan taxa [68,69]. Our development of a genetic map for coral, which, to our knowledge, constitutes the first genetic map for a non-bilaterian metazoan, reveals the conservation of genomic organization among distantly related animal taxa.

As the simplest free-living animals, placozoans represent a primitive metazoan form. A recent comprehensive phylogenetic study suggests that Placozoa are basal relative to all other non-Bilaterian animals ([70], but see [71]). Whole genome analysis of placozoan *T. adhaerens *shows that the placozoan genome has the lowest amount of local rearrangement relative to the common placozoan-cnidarian-bilaterian ancestor [69]. Previous comparative genome analysis revealed synteny blocks shared between placozoan and human genomes, which likely reflect ancestral features of the metazoan genome. In our study, we also found extensive synteny between coral and placozoan genomes (despite the incomplete assembly of the placozoan genome), suggesting that the coral genome also preserves many features of ancestral genome organization.

Our preliminary synteny analysis identified numerous synteny blocks in each comparison between the coral map and other metazoan genomes. However, because of the number and positions of markers and their matching sequences within the two genomes, a substantial number of synteny blocks could be expected to arise by chance. Several methods to test for significant evidence of synteny between two genomes, based on randomly shuffled permutations of the real data, have been previously described [72-75]. The existing implementations of these methods are not well suited for our data (comparison of genetic maps and genome sequences across distantly related taxa), but are more applicable to comparative genome analysis of closely related species [19], because they require marker colinearity (that is, conserved marker order), and/or assume chromosome homology between chromosomes in comparison (for example, randomize markers only within a chromosome to evaluate significance of identified synteny). We followed a similar approach for our analysis, by randomly shuffling marker positions across the entire map and evaluating the likelihood that the number of synteny blocks, and the number of markers in each block, could have arisen by chance.

Without any statistical tests, a simple analysis of synteny could be easily misinterpreted; for example, the large number of synteny blocks found in comparisons between the coral map and the worm and fly genomes (12 to 13 blocks in each comparison, with 3 to 10 markers per block) might suggest that the coral genome shared more structural similarities with worm and fly than with other animal genomes. However, permutation tests revealed that, in fact, neither of those comparisons found more synteny blocks than expected by chance (Table 3). There are several characteristics of genomic structure that would obviously be expected to affect the detection of synteny blocks by our criteria, including genome size, chromosome numbers, and the completeness of the assembly. Because the genomes considered in this study differed widely in these characteristics, this posed an important caveat for any conclusions drawn from these comparisons. Importantly, each of the comparisons between the coral map and another metazoan genome included at least one block that was significantly larger than expected by chance, based on permutation tests of block size (the number of markers within each block).

### Maintenance of synteny across great evolutionary distances

If not maintained by natural selection, synteny would be expected to break down between distantly related taxa. One obvious factor that would affect this is the rate of genome rearrangement. Recent studies have shown that rates of chromosomal rearrangement are much higher in invertebrates than vertebrates [76-78]. For example, the rearrangement rates of *Drosophila *and *Caenorhabditis *are 350 to 850 and 1,400 to 17,000 times higher than those of mammals, respectively [77]. Our finding that the coral map and the worm and fly genomes share very little conserved synteny is consistent with these previous reports. Still, the worm and fly genomes do contain a small number (one and two, respectively) of synteny blocks (each including nine to ten genes), and these are significantly larger than expected by chance (Table 3).

In general, eukaryotic genomes evolve by random micro- and macro-rearrangements such as indels, inversions and translocations [79]. Nevertheless, gene distribution in eukaryotic genomes is not random [80]. Several hypotheses have been put forward to explain synteny. Early research in genomic evolution and synteny assumed no selection for synteny, and suggested that synteny resulted from ancestral linkage groups that had not yet been disrupted by random chromosomal rearrangements [81]. The subsequent discovery that certain groups of co-regulated genes showed strict conservation of both gene order and linkage across taxa [82] refined this model by demonstrating that the co-regulation of a group of genes by local regulatory elements can drive conservation of synteny blocks containing those genes and their corresponding regulatory elements [83]. Recent studies have suggested an additional mechanism driving the conservation of synteny: the interdigitation of regulatory elements and their target genes by other genes with unrelated functions and regulatory pathways [84,85].

None of those proposed mechanisms provides a clear explanation for our findings. Several metazoan genomes showed more synteny blocks than expected by chance, but the gene functions suggested by sequence similarity for these syntenic markers were not linked in any obvious way. For example, the map includes one pair of genes that is linked in three species: LG5 of coral, chromosome 5 of worm, and chromosome 14 of human ([GenBank:EZ001917] and [GenBank:EZ012107]; Additional data file 1). There is no clear functional relationship between the genes associated with these markers (serine palmitoyltransferase 2, and enhancer of rudimentary homolog). Obviously this does not preclude the possibility of unknown functional relationships among the mapped genes, or of functional relationships between the other genes not included in the coral map. The list of syntenic markers associated with known genes also did not include any known examples of co-regulated genes (Additional data file 1). The identification of synteny blocks from the coral genetic map therefore provides no support for either explanation, but raises a number of interesting questions. Synteny blocks were distributed differently among taxa; for example, both fly and placozoan genomes showed conserved synteny with regions of LG2, but only the placozoan genome did with LG4 (Figure 5). The extent to which these differences are explained by selective pressures versus rates of genome rearrangements (for example, [77]) is not clear from our data, but this will probably become a more tractable question as genome sequences become available for a broader sampling of metazoan taxa. The extensive rearrangements evident within synteny blocks in the coral map (Figure 5) prompt questions about what mechanisms might account for conserved linkage but highly variable order. We speculate that selection might promote linkage between genes that must be modified in a correlated fashion to achieve an adaptive advantage (in other words, exhibit epistatic interactions). Linkage between epistatically interacting loci would allow for selection to operate on haplotypes rather than individual alleles [86], which would substantially improve the heritability of the evolving trait and hence the efficiency of selection. There are several pan-metazoan systems that can be viewed in terms of many correlated (or anti-correlated) traits determined by genes with otherwise unrelated functions. Examples include epithelial functions (rigidity, across-epithelial transport, along-epithelial connectivity, cuticle secretion, ciliation), cell-cell communication and nutrient exchange, and organism-wide transport and excretion. Future comparative analysis of genome sequence and function in the basal metazoans like *A. millepora *may help to elucidate the evolutionary origin of the pan-metazoan synteny.

## Conclusions

A genetic linkage map, predominantly based on SNP markers derived from the transcriptome, has been constructed for a reef-building coral, *Acropora millepora*. This map has ample resolution for QTL analysis (3.4 cM) and represents the first linkage map for a coral, as well as for any non-bilaterian multicellular organism. The map will become the foundation for QTL analysis of adaptive traits and population genomics in the coral, to address the problem of coral evolution response to climate change, as well as for coral genome assembly. Comparative genomic analysis based on this map revealed a few statistically significant synteny blocks, which may reflect the features of ancestral metazoan genome organization. The specific mechanisms underlying such preservation are not yet clear, but represent an exciting area for future studies.

## Materials and methods

### Coral mapping family

A full-sibling family was established by crossing of two colonies of *A. millepora*, which were collected at Magnetic Island, Queensland, Australia, in 2007. One of the colonies served as a male parent (that is, only contributed sperm to the cross), while the other contributed eggs and served as a female parent. The procedures of fertilization and larval culture are described in [11]. In an effort to use the same material for expression QTL mapping of heat tolerance in future, larvae were reared at an elevated temperature of 32°C rather than a standard culturing temperature (for example, 28°C). Parental sperm and 5-day post-fertilization larvae were preserved in pure ethanol and RNALater (Ambion, Foster City, CA, USA), respectively, for genotyping. In total, 80 larvae were used for linkage mapping analysis.

### DNA extraction and whole-genome amplification

Parental DNA was extracted from the preserved sperm using DNeasy Blood & Tissue kit (Qiagen, Valencia, CA, USA). We have developed a protocol for parallel extraction of DNA and RNA from single coral larva. Each larva was incubated at room temperature in 100 μl lysis solution from the RNAqueous kit (Ambion, Foster City, CA, USA) for 10 minutes and then centrifuged at 16,000 G for 5 minutes. Supernatant was transferred for total mRNA extraction using the RNAqueous-Micro kit (Ambion, Austin, TX, USA). The remaining pellet of cell debris was washed with 100 μl 1× phosphate-buffered saline, which was discarded after centrifuging at 16,000 G for 2 minutes, and then digested in 100 μl digest buffer (100 mM NaCl, 10 mM Tris-Cl (pH 8.0), 25 mM EDTA (pH 8.0), 0.5% SDS and 0.1 mg/ml proteinase K) at 42°C for 2.5 hours. After digestion, the solution was centrifuged at 4,000 G for 2 minutes, and supernatant was transferred into a new tube. Then 80 μl 100% isopropanol was added to the supernatant in order to precipitate larval DNA. The solution was held at -20°C for 30 minutes and then centrifuged at 4°C for 20 minutes at 16,000 G. The resulting DNA pellet was washed using 200 μl of 40% isopropanol, which was discarded after centrifuging at 4°C for 5 minutes at 16,000 G. After air-drying the pellets, DNA was dissolved in 15 μl elution buffer (Qiagen).

To make sufficient DNA templates for several hundred PCR amplifications, we used the REPLI-g Mini kit (Qiagen) for whole-genome amplification of larval DNA samples. For each larva, approximately 10 ng larval DNA was used as input DNA for whole-genome amplification. The REPLI-g Mini kit utilizes a Phi29 DNA polymerase-based multiple displacement amplification technique, which can produce high fidelity and near-complete genome representation suitable for high resolution SNP genotyping [87-89].

### Microsatellite genotyping

Fifty microsatellite markers were genotyped in this study, of which 40 were developed by our group [8] and 10 were from [9]. For each marker, one of the two primers used was fluorescently labeled with 6-carboxyfluorescein or hexachlorofluorescein. PCR amplification and fragment analysis by capillary electrophoresis followed the same procedure as described in [8].

### SNP marker development, genotyping and inter-populationtransferability

More than 33,000 candidate SNPs were previously identified in the *A. millepora *larval transcriptome by sequence analysis [11]. Of these, 1033 were selected for marker development using the criteria of at least 3× occurrence of the minority allele and at least 6× read coverage. Most of the SNP markers were named as follows: C followed by several numbers refers to a CAP3-assembled contig number, and then S followed by several numbers refers to the SNP position (bp) in this contig. In addition, four SNP markers were developed from introns, so they were named only by the contig number. We have developed a cost-effective method for SNP genotyping using the HRM capability of the Roche (Indianapolis, IN, USA) LightCycler 480. For one SNP assay, three unmodified oligonucleotides were used, which corresponded to two PCR primers and one probe. Each SNP locus was first amplified by an asymmetrical PCR (1:5 in primer concentration) with HRM fluorescent dye in the PCR master mix and was then interrogated by an unlabeled probe with two mismatched bases at its 3' end. Primers were designed based on several principles as described in [90] so that all PCR amplifications could be achieved at the same annealing temperature. In an effort to decrease the chance of amplifying introns, the expected amplicon lengths were usually restricted to about 100 bp. Probes were designed according to the following rules: T_m _of approximately 60°C; probe length between 20 and 35 bases; SNP sites located near the middle of each probe to maximize the instability with a mismatched variant; and two mismatched bases added to the 3' end of each probe to prevent extension. PCR amplifications were performed in 384-well plates in a 15-μl volume composed of approximately 20 ng amplified genomic DNA, 0.1 μM forward primer, 0.5 μM reverse primer, 2 mM MgCl_2_, and 1× HRM Master Mix (Roche) in the Roche LightCycler 480 instrument. All cycling began with an initial denaturation at 95°C for 10 minutes, followed by 65 cycles of 95°C for 40 s, 60°C for 40 s, and 72°C for 40 s. For primer testing, 1 μl of PCR product was run on a 1.5% agarose gel to determine the success of the PCR. After PCR amplification, an aliquot of the appropriate probe was added in each reaction to a final concentration of 5 μM. HRM genotyping was performed on the Roche LightCycler 480 instrument with an initial denaturation at 95°C for 1 minute, cooling at a rate of 2.5°C/s to 40°C with a 1-minute hold, and then continuous melting curve acquisition (25 acquisitions per °C) during a 0.02°C/s ramp to 95°C. Data were retrieved and analyzed using the LightCycler 480 Software 1.5, with manual curation of genotype calls. The primer and probe sequences for all mapped markers are available in Additional data file 1. To evaluate the inter-population transferability of our SNP markers, seven *A. millepora *colonies were tested using 48 randomly chosen SNP markers, of which four came from the Orpheus Island and three from the Great Keppel Island, which are 80 km (NNW) and 570 km (SSE) away from Magnetic Island, respectively.

### Linkage analysis

Linkage analysis was carried out using JoinMap 4.0 software [23]. Genotype configurations of markers were categorized into four types with null-allele allowed: 1:1:1:1 type (female × male: AB × CD or AB × AC), 1:2:1 type (AB × AB), 1:1 female type (AB × AA or CC), and 1:1 male type (AA or CC × AB). For all segregating loci, goodness-of-fit of the observed with expected Mendelian ratios were assessed with chi-square test. A LOD score of 4.5 was initially set as the linkage threshold for grouping markers. Once 14 linkage groups corresponding to the known haploid chromosome number for this species were determined, the rest of the markers were added to their corresponding groups using a less stringent criterion of LOD ≥2.5. Sex-specific maps were first constructed for each parent using the two-way pseudo-testcross strategy [24]. Maternal (1:1 female type) and paternal (1:1 male type) datasets were created using the function of 'Create Maternal and Paternal Population Nodes' in the JoinMap program, which also partitioned 1:1:1:1-type data into 1:1 female- and 1:1 male-type data, but ignored 1:2:1-type data. The JoinMap program uses *G*^2 ^statistic for independence test. The power of this statistic in determining marker linkage is not influenced by segregation distortion [23,46]. The regression mapping algorithm was used for map construction, which is a procedure of building a map by adding loci one by one when starting from the most informative pair of loci [91]. The best fitting position of an added marker was searched on the basis of goodness-of-fit test for the resulting map. To prevent being trapped in a local optimum of the goodness-of-fit, 'ripple' was performed each time after adding one locus. The Kosambi mapping function [92] was used to convert the recombination frequencies into map distance (centiMorgans). Once the female and male maps were established, a consensus map was constructed using markers with all genotype configurations (the 'CP' population model). Marker orders in the female and male maps were used as preferred orders (the 'fixed orders' function) in the consensus map construction. MapChart 2.2 software [93] was used for graphical visualization of the linkage groups.

### Map length and coverage

On the basis of the consensus map, we used two methods to calculate estimated genome length. The first estimator (*G*_*e*1_) was calculated by adding 2s to the length of each linkage group to account for chromosome ends [27], where s is the average spacing between markers, which was calculated by dividing the total length of all linkage groups by the number of intervals (number of markers minus number of linkage groups). The second estimator (*G*_*e*2_) was calculated by multiplying the length of each linkage group by (m + 1)/(m - 1) [28], where m is the number of markers in that linkage group. Genome coverage was estimated by *G*_*o*_/*G*_*e*_, where *G*_*o *_is the observed genome length and *G*_*e *_is the average of *G*_*e*1 _and *G*_*e*2_.

### Comparative genome analysis

To enable sequence-based comparisons between the coral genetic map and other eukaryote genomes, we associated each of the markers mapped in this study with a previously annotated cDNA sequence from the coral transcriptome [11]. The annotated sequence corresponding to each marker-containing contig was identified by blastn, with a significance threshold of bit-scores ≥100. Bit-scores were used instead of e-values because of the effects of the small database size on e-values. The markers that matched annotated genes [11] were assigned gene names based on those annotations. The mapped cDNA sequences identified by this process were longer (average = 1,232 bp) than the working CAP3-derived contigs from which the markers were identified (average = 711 bp), and these longer sequences were used for all subsequent comparisons between the coral genetic map and genomic sequences from other organisms.

Mapped coral sequences were compared with other eukaryotic genomes using tblastx (bit-scores ≥50) to identify regions of synteny (the conserved linkage of genes in different genomes). This analysis included high-quality assemblies for three bilaterian animals: release 4 of the human reference genome assembly, release 5.17 for *D. melanogaster*, and release WS202 for *C. elegans*. Draft assemblies for two non-bilaterian animals were included to provide a broader taxonomic comparison: assembly 1.0 for the cnidarian *N. vectensis *and assembly 1.0 for the placozoan *T. adhaerens*. The reference yeast genome assembly of *S. cerevisiae *was also included as a representative non-metazoan eukaryote.

Synteny blocks conserved between the genomes of coral and other animals were identified based on the position of each mapped coral sequence within a linkage group, and the position of its best match in the other genome. For each pairwise comparison (tblastx) between mapped coral sequences and the other species' genome sequences, we identified synteny blocks based on the following operational criteria. First, blocks were required to contain three or more markers within a single linkage group in the coral map, and those markers had to match three regions within a single chromosome or scaffold in the other genome. Second, the nearest neighbor for each marker within the coral map had to be within ≤10 cM. Third, the nearest neighbor for each of the matches in the other genome had to be within ≤10 Mb. Application of these criteria identified numerous blocks of genes that showed conserved linkage across genomes, including blocks with substantial intra-chromosomal rearrangements.

We used permutation tests to evaluate the significance of the observed synteny. First, marker labels and positions in the coral map were shuffled for each permutation (n = 1,000), using the shuffle subroutine of the List::Util module in Perl. Within each randomly shuffled dataset, the number of synteny blocks and the numbers of markers in each block that emerged by chance were tabulated. The probability that the observed synteny blocks (that is, in the original, non-shuffled data) resulted from random chance was calculated from the percentage of shuffled datasets that produced at least as many synteny blocks as the original data. This provided an estimate for the significance of each between-genome comparison. These permutations also allowed us to estimate the significance of particular block sizes within each comparison (for example, to evaluate the probability that a block of ten markers with conserved linkage resulted from random chance). For each block size within a particular comparison, based on the number of markers included in that block, we calculated the probability that a block that large would emerge by random chance based on the percentage of blocks that large or larger in the randomly shuffled datasets. This provided an estimate for the significance of each block size within each comparison.

## Abbreviations

HRM: high-resolution melting; LOD: logarithm of the odds; QTL: quantitative trait locus; SNP: single nucleotide polymorphism.

## Competing interests

The authors declare that they have no competing interests.

## Authors' contributions

MVM conceived the study. EM and MVM performed the cross and larval rearing. SW and LZ conducted DNA preparation, SNP and microsatellite genotyping, and linkage analysis. EM conducted comparative genome analysis. SW, EM and MVM drafted the manuscript. All authors read and approved the final manuscript.

## Additional data files

The following additional data are available with the online version of this article: an Excel table containing detailed information of mapped SNP markers (primer and probe sequences, gene annotation and synteny) (Additional data file 1).

## Supplementary Material

Additional data file 1 Detailed information of mapped SNP markers (primer and probe sequences, gene annotation, and synteny). Click here for file
